# Susceptibility of BAFF-var allele carriers to severe SLE with occurrence of lupus nephritis

**DOI:** 10.1186/s12882-019-1623-4

**Published:** 2019-11-21

**Authors:** Justa Friebus-Kardash, Marten Trendelenburg, Ute Eisenberger, Camillo Ribi, Carlo Chizzolini, Uyen Huynh-Do, Karl Sebastian Lang, Benjamin Wilde, Andreas Kribben, Oliver Witzke, Sebastian Dolff, Cornelia Hardt

**Affiliations:** 1 Department of Nephrology, University Hospital Essen, University of Duisburg-Essen, Hufelandstraße 55, 45147 Essen, Germany; 2 Institute of Immunology, Medical Faculty, University of Duisburg-Essen, Hufelandstraße 55, 45147 Essen, Germany; 3 Division of Internal Medicine and Clinical Immunology Laboratory, Department of Biomedicine, University Hospital, Spitalstraße 21, 4031 Basel, Switzerland; 4 Immunology and Allergy, Department of Internal Medicine, University Hospital, Rue du Bugnon 46, 1005 Lausanne, Switzerland; 5 Immunology and Allergy, Department of Internal Medicine, University Hospital and School of Medicine, Rue Gabrielle-Perret-Gentil 4, 1205 Geneva, Switzerland; 6 Division of Nephrology and Hypertension, University Hospital, Freiburgstraße 18, 3010 Bern, Switzerland; 7 Department of Infectious Diseases, University Hospital Essen, University of Duisburg-Essen, Hufelandstraße 55, 45147 Essen, Germany

**Keywords:** BAFF-var allele, TNFSF13B, Systemic lupus erythematosus, Lupus nephritis, Disease activity, Swiss SLE cohort study (SSCS)

## Abstract

**Background:**

Dysregulation of the B-cell activating factor (BAFF) system is involved in the pathogenesis of systemic lupus erythematosus (SLE). Increased serum concentrations of BAFF are related to lupus nephritis and disease activity among SLE patients. Recently, a variant of the BAFF-encoding gene, BAFF-var, was identified to be associated with autoimmune diseases, in particular SLE, and to promote the production of soluble BAFF. The present study aimed to assess the prevalence of BAFF-var in a cohort of 195 SLE patients and to analyze the association of the BAFF-var genotype (TNSF13B) with various manifestations of SLE.

**Methods:**

A cohort of 195 SLE patients from Central Europe, including 153 patients from the Swiss SLE Cohort Study and 42 patients from the University Hospital Essen, Germany, underwent genotyping for detection of BAFF-var allele.

**Results:**

Of the 195 patients, 18 (9.2%) tested positive for BAFF-var variant according to the minor allele frequency of 4.6%. The presence of BAFF-var was associated with the occurrence of lupus nephritis (*p* = 0.038) (*p* = 0.03 and *p* = 0.003). Among various organ manifestations of SLE, the presence of BAFF-var was associated with the occurrence of lupus nephritis (*p* = 0.038; odds ratio [OR], 2.4; 95% confidence interval [CI], 0.89–6.34) and renal activity markers such as proteinuria and hematuria (*p* = 0.03; OR, 2.4; 95% CI, 0.9–6.4 for proteinuria; *p* = 0.003; OR, 3.9; 95% CI, 1.43–10.76 for hematuria). SLE patients carrying the BAFF-var allele exhibited increased disease activity at study entry, as determined by the physician’s global assessment (PGA: *p* = 0.002; OR, 4.8; 95% CI, 1.54–14.93) and the SLE Disease Activity Index (*p* = 0.012; OR, 3.5; 95% CI, 1.12–11.18). Consistent with that, the percentage of patients treated with immunosuppressive agents at study entry was higher among those carrying the BAFF-var allele than among those tested negative for BAFF-var (*p* = 0.006; OR, 3.7; 95% CI, 1.27–10.84).

**Conclusions:**

Our results indicate an association between the BAFF-var genotype and increased severity of SLE. Determining the BAFF-var status of SLE patients may improve the risk stratification of patients for whom the development of lupus nephritis is more likely and thus may be helpful in the follow-up care and treatment of SLE patients.

## Background

Systemic lupus erythematosus (SLE) is an autoimmune disorder that, if left untreated, can cause irreversible damage to multiple tissues and organ systems. Renal involvement, so-called lupus nephritis, is one of the most frequent and severe organ manifestations of SLE [1, 2]. Lupus nephritis is considered to be the consequence of a heterogeneous spectrum of autoantibodies that are a serological hallmark of SLE [3, 4]. The most widely recognized mechanism of organ injury mediated by autoantibodies is the formation of circulating immune complexes associated with activation of the complement system [5]. However, various environmental factors on a disease-prone genetic background appear to trigger development of unrestricted hyperactivation of the immune system, resulting in the onset of SLE [6].

In recent years, the treatment of SLE has evolved from conventional drugs providing a general immunosuppressive function to novel biological-targeting strategies, of which belimumab is an important and successful therapeutic agent [7, 8]. Belimumab has been approved as a treatment for SLE and is a human monoclonal antibody directed against soluble B-cell activating factor (BAFF). BAFF was considered to contribute to the pathogenesis of SLE by enhancing the proliferation and survival of autoreactive B cells, thereby leading to overproduction of diverse autoantibodies [9, 10]. In support of this theory, abnormal serum levels of BAFF have been detected among SLE patients [10, 11]. Our previous study and numerous other reports found an association between BAFF serum concentrations, disease activity, and the occurrence of lupus nephritis [11–14]. In line with these observations, clinical trials demonstrated the effectiveness of belimumab in reducing the number of autoantibodies and reducing disease activity among SLE patients [8, 15, 16].

One possible reason for increased BAFF levels in SLE patients seems to be genetic alterations. Thus, Steri et al. described for the first time a functional genetic variant of the BAFF encoding gene, tumor necrosis factor ligand superfamily member 13b (TNFSF13B), which causes overexpression of soluble BAFF and is associated with an increased risk of autoimmunity [17]. BAFF-var (a variant of TNFSF13B) is generated by an insertion-deletion characterized by the replacement of five nucleotides, GCTGT, by one, A (GCTGT→A). The BAFF-var allele yields an alternative polyadenylation signal, which in turn results in a shorter mRNA transcript. The binding sites for the inhibitory microRNA are missing from this short mRNA transcript, and their absence prevents downregulation of BAFF expression [17]. In addition the RNA-binding protein, nuclear factor 90 (NF90), was also found to bind selectively to the BAFF mRNA and to cooperate with the microRNA-15a in order to suppress the further BAFF translation [18]. In case of occurrence of the BAFF-var allele the inhibitory action of NF90 on the BAFF translation is omitted due to the lack of binding sites for NF90 on the short mRNA transcript. Accordingly, heterozygous carriers of the genetic variant, BAFF-var, exhibit increased production of circulating BAFF, larger numbers of B cells, and higher concentrations of immunoglobulins. The prevalence of BAFF-var is notably elevated among patients with autoimmune diseases, such as multiple sclerosis, SLE, and rheumatoid arthritis. This fact implies a link between BAFF-var variant and susceptibility to autoimmune processes [17, 19]. However, to date the relationship between BAFF-var and SLE disease activity in specific organs has not been elucidated.

The goals of the current study were to investigate the prevalence of BAFF-var among our central-European SLE cohort consisting of 195 patients and to analyze the association between BAFF-var and the typical clinical and laboratory features of SLE.

## Methods

### Study population

The study population consisted of 195 adult patients with SLE. All study subjects met at least 4 of 11 classification criteria endorsed by the American College of Rheumatology. We obtained 153 samples of whole blood from SLE patients who were prospectively enrolled in the Swiss SLE Cohort Study (SSCS) and 42 samples of whole blood from SLE patients recruited at the University Hospital Essen. The samples were collected between 2007 and 2018 and were stored at − 80 °C before DNA was isolated. Corresponding clinical and laboratory data obtained at the time of entry into the SSCS or into the Essen cohort of SLE patients were used for the further analysis. Disease activity was estimated by the SLE Disease Activity Index (SLEDAI) and the Physician’s Global Assessment (PGA) for the 153 patients from the SSCS and by the SLEDAI alone for the 42 patients from the University Hospital Essen. Patients with a SLEDAI score lower than 6 points were considered to have inactive or minimally active disease [20, 21]. Patient characteristics of the current cohort are presented in Table 1.

**Table 1:**
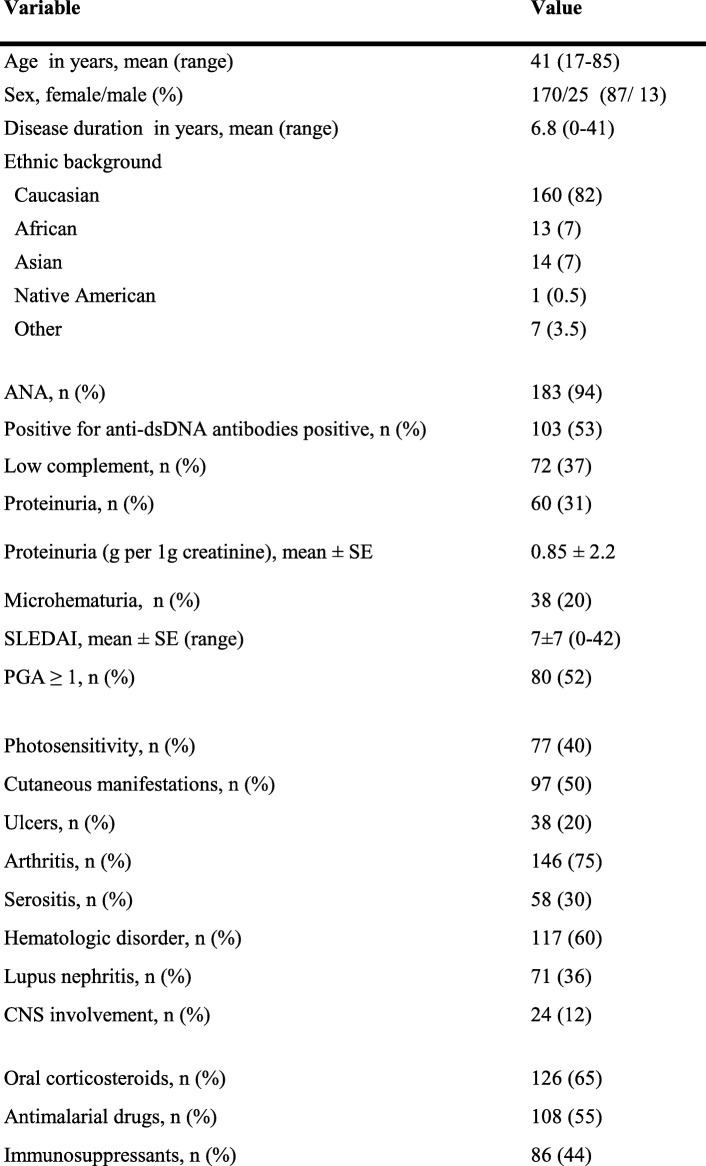
Demographic characteristics of 195 patients with systemic lupus erythematosus

*ANA* antinuclear antibodies; *CNS* central nervous system; *dsDNA* double-stranded DNA; *PGA* physician’s global assessment; *SLEDAI* Systemic Lupus Erythematosus Disease Activity Index

The study was approved by the local ethics committees of the participating centers (University Hospital Geneva, 06–100; University Hospital Lausanne, 04/07; University Hospital Bern, KEK-BE 045/07; University Hospital Basel, EKBB 262/06; University Hospital Essen, 12–5149-BO). All patients signed a written informed consent.

### Genotyping

Genomic DNA was isolated from 153 whole-blood samples from SSCS using the QIAamp DNA Blood Midi Kit (QIAGEN GmbH, Hilden, Germany) according to the manufacturer’s instructions. For the 42 SLE patients from the University Hospital Essen, DNA was purified from peripheral blood leukocytes as previously described [22]. For polymerase chain reaction (PCR), 50 ng of genomic DNA was amplified in a reaction tube containing 5 pmol of each primer (BAFFVRF, 5′-CTCAGAAGACAGCATCCCGGT-3; BAFFVRR, 5′-GAGAAGAATGCTTGGCCTGCT-3), 1x reaction buffer, 1 U DNA Taq DNA Polymerase (QIAGEN GmbH, Hilden, Germany) and 200 μM of deoxyribonucleotide triphosphate (dNTP Mix; Promega GmbH, Mannheim, Germany). Cycling parameters were as follows: initial denaturation at 94 °C for 2 min, 35 cycles at 94 °C for 15 s, annealing at 60 °C for 30 s, at 72 °C for 1 min, and a final extension step at 72 °C for 7 min. PCR products were analyzed by electrophoresis in a 1.5% agarose gel stained with ethidium bromide for identifying the BAFF-specific products (890 bp) for use as a template in sequencing reactions. PCR samples were purified with the QIAquick 96 PCR Purification Kit (QIAGEN GmbH, Hilden, Germany). Sequencing reactions were performed with the BAFFVRS –5′-TCAGATATGGAACATACTCACAT-3 primer and the BigDye Terminator™ v1.1 Cycle Sequencing Kit (Thermo Fisher Scientific, Schwerte, Germany), essentially as described by the manufacturer. Sequencing products were run on an ABI 3130XL Genetic Analyzer (Applied Biosystems, Foster City, CA, USA). Data analysis was performed with Chromas software version 2.6.5 (Technelysium, South Brisbane, Australia).

### Statistical analysis

Data are given as absolute numbers and percentages or as means ± standard deviation. For comparisons of two groups one-tailed Chi-square tests were carried out. The level of statistical significance was set at *p* = 0.05. Statistical analyses were performed with GraphPad Prism version 6 (GraphPad Software, Inc., San Diego, CA, USA).

## Results

### Patient characteristics

**Fig. 1:**
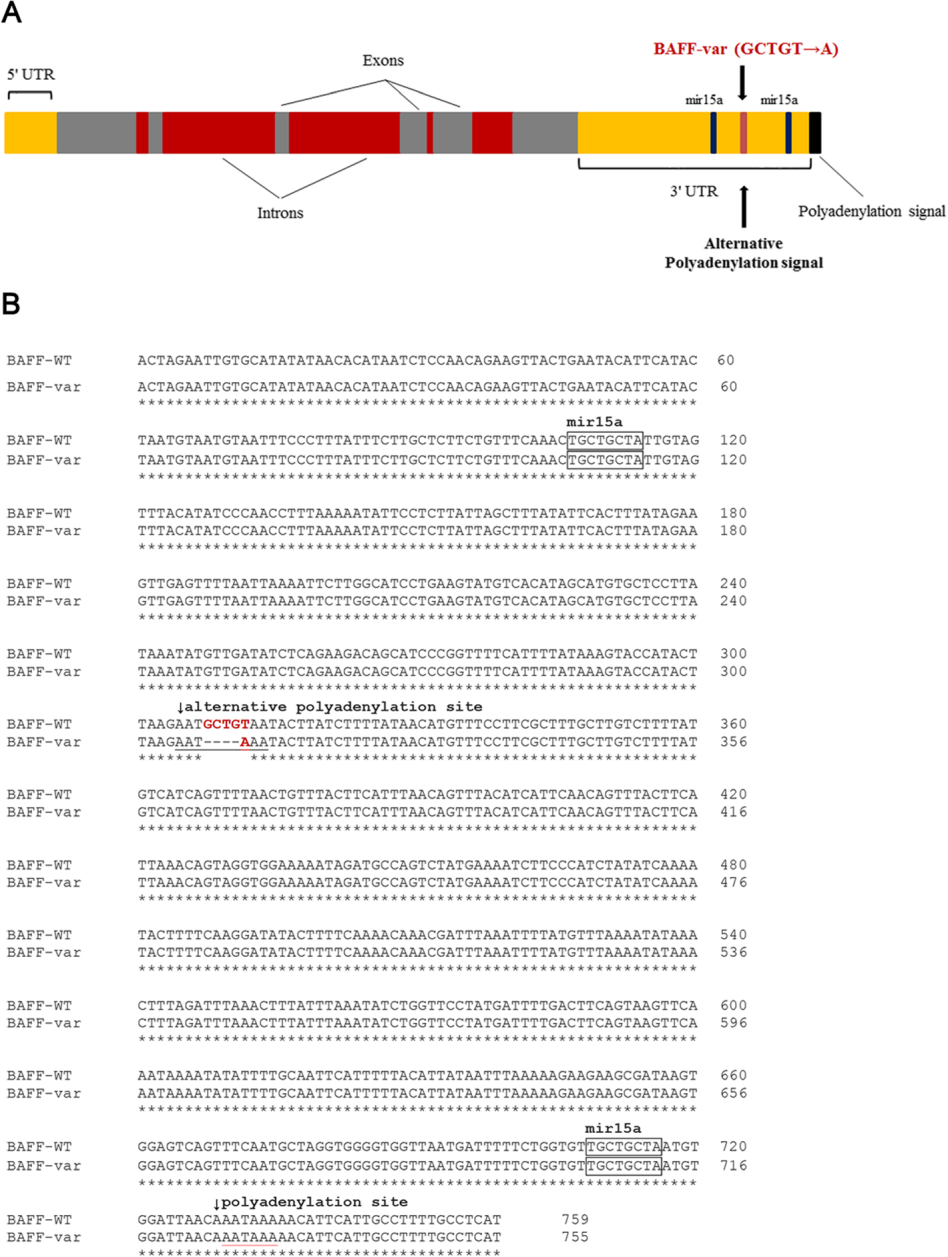
Location of the BAFF-var within the TNFSF13B gene encoding BAFF. **a** Schematic illustration of the location of BAFF-var in the three prime untranslated region of TNFSF13B. **b** Nucleotide sequence alignment of the BAFF-var and wildtype allele. UTR, untranslated region; mir15a, micro ribonucleic acid-15a

We determined the prevalence of the TNFSF13B gene variant, BAFF-var, in our central-European cohort of 195 SLE patients, including 153 patients from the SSCS and 42 patients from the University Hospital Essen (Fig. 1). Demographic and clinical data of the study cohort are summarized in Table 1. Most of our patients were of Caucasian descent; 13 of the 195 patients (7%) were Africans; and 14 (7%) were Asian. With regard to manifestations of SLE in various organs or organ systems at the time of study entry, 75% of patients had arthritis and 60% had hematological alterations. At study entry, a biopsy proven lupus nephritis was observed in 71 patients corresponding to 36% of the total cohort. Among the 71 patients, 30 patients had lupus nephritis class IV, 10 patients had class III and 13 patients had class V. While lupus nephritis class II was present in 13 patients and class I in one patient, respectively, lupus nephritis class was unknown in 4 patients. Clinical signs of renal manifestation such as proteinuria and hematuria were present in 60 (31%) and 38 patients (20%), respectively. Approximately half of our SLE cohort tested positive for anti–double stranded DNA (anti-dsDNA) antibodies, and 72 subjects (37%) exhibited complement consumption defined as low complement factor 3 and/or low complement factor 4. Two scoring systems reflecting the disease activity of SLE, the SLEDAI and the PGA, were determined for all 153 patients from the SSCS, whereas the SLEDAI alone was used for the 42 patients from the University Hospital Essen. The mean SLEDAI score of the total study population estimated at the time of study entry was 7 (±7 (0–42)), indicating moderate disease activity. At inclusion, more than half of patients were undergoing treatment with antimalarial agents, and 44% required treatment with immunosuppressive agents. The immunosuppressants were cyclophosphamide, azathioprine, mycophenolate mofetil, mycophenolic acid, cyclosporine A, methotrexate, leflunomide and rituximab.

### BAFF-var genotype in SLE patients

The BAFF-var allele was detected in 18 of our 195 SLE patients (9.2%); all 18 were heterozygous for the BAFF-var allele. The minor allele frequency (MAF) of BAFF-var was 0.0462 (4.6%). There was no significant difference in ethnic background between the 18 patients heterozygous for the BAFF-var allele and the 177 patients who did not carry this allele (data not shown). Of the 18 SLE patients carrying the BAFF-var allele, 16 were Caucasians and one was of African origin. Another patient, from South Africa, was of mixed origin.

### Association of BAFF-var allele with lupus nephritis and severity of SLE

With regard to diverse manifestations of SLE at the time of study entry, a biopsy proven lupus nephritis developed in 10 of the 18 patients (56%) with the BAFF-var allele and in 61 of the 177 patients (34%) who did not carry this allele (*p* = 0.038; OR [odds ratio], 2.4; 95% confidence interval [CI], 0.89–6.34) (Table 2). Although the frequency of lupus nephritis was significantly higher among patients carrying the BAFF-var allele, there were no important differences between the two groups with regard to other manifestations of SLE and the involvement of other organs, such as inflammatory skin manifestations, photosensitivity, ulcers, arthritis, serositis, hematological disorders, or central nervous system involvement (Table 2). At study entry, 9 of the 18 patients being heterozygous for BAFF-var (50%) had a proteinuria occurring in the context of renal manifestation of SLE that was higher than 0.3 g per 1 mg urine-creatinine, whereas only 51 (29%) of the 177 SLE patients without the BAFF-var allele had proteinuria (*p* = 0.03; OR, 2.4; 95% CI, 0.9–6.4) (Table 3). Hematuria associated with active lupus nephritis also occurred more frequently among carriers of the BAFF-var polymorphism (8 of 18, 44%) than among patients without this polymorphism (30 of 177, 17%) (*p* = 0.003; OR, 3.9; 95% CI, 1.46–10.76) (Table 3). As shown in Table 3, there were no significant differences between the study groups with regard to the presence of antinuclear antibodies (ANA), anti-dsDNA antibodies, or hypocomplementemia.

**Table 2:**
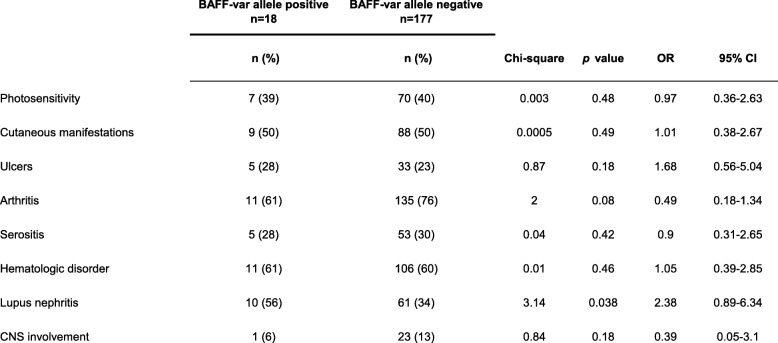
Association of the BAFF-var allele with various organ manifestations in 195 patients with systemic lupus erythematosus

*CI* confidence interval; *CNS* central nervous system; *OR* odds ratio

**Table 3:**
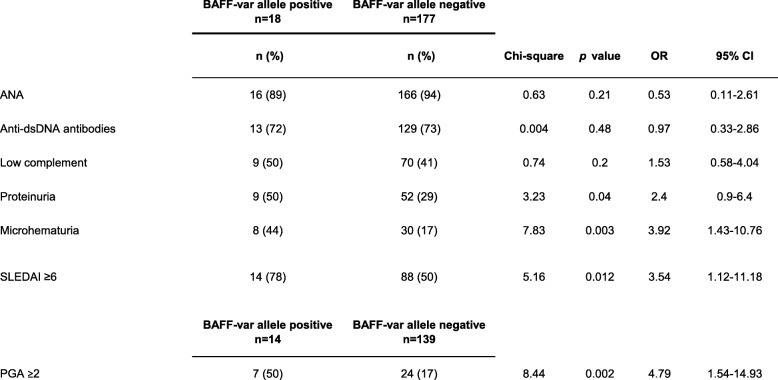
Association of the BAFF-var allele with routine serological parameters and disease activity of systemic lupus erythematosus among 195 patients

*ANA* antinuclear antibodies; *CI* confidence interval; *dsDNA* double stranded DNA; *OR* odds ratio; *PGA* physician’s global assessment; *SLEDAI* Systemic Lupus Erythematosus Disease Activity Index

Taking into account disease activity of SLE at the time of inclusion, patients with the BAFF-var allele exhibited severe disease as assessed by the attending physician (and subsequently defined as PGA ≥ 2) more frequently (7 of 14, 50%) than did those without this allele (24 of 139, 17%) (*p* = 0.002; OR, 4.8; 95% CI, 1.54–14.93) (Table 3). In addition, 14 of 18 (78%) patients carrying the BAFF-var allele and 88 of 177 patients (50%) negative for BAFF-var allele had a SLEDAI score of 6 or higher, defined as moderate to high disease activity (*p* = 0.012; OR, 3.5; 95% CI, 1.12–11.18) (Table 3). Consequently, the frequency of patients receiving immunosuppressive agents at the time of study entry was significantly higher among carriers of the BAFF-var allele (13 of 18, (72%) than among patients without this allele (73 of 177, 41%) (*p* = 0.006; OR, 3.7; 95% CI, 1.27–10.84) (Table 4). Moreover, the frequency of administration of a combination of antimalarial drugs and immunosuppressive agents was higher for the SLE patients carrying the BAFF-var allele (8 of 18, 44%) than for those without this allele (35 of 177, 20%) (*p* = 0.008; OR, 3.3; 95% CI, 1.19–8.83), as shown in Table 4. The frequencies of administration of steroids and antimalarial drugs alone were similar between patients carrying the BAFF-var genotype and those not carrying it (Table 4).

**Table 4:**
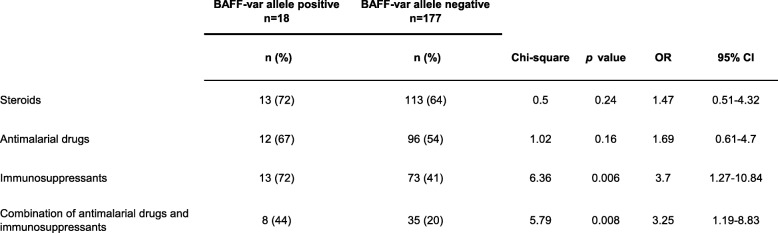
Association of the BAFF-var allele with therapy being administered to 195 patients with systemic lupus erythematosus at study entry

*CI* confidence interval; *OR* odds ratio

## Discussion

This study found that the prevalence of the TNFSF13B BAFF-var among German and Swiss SLE patients was 9.2%. Patients with the BAFF-var genotype were more likely to exhibit signs of renal involvement, such as lupus nephritis, proteinuria, and hematuria. Compared to SLE patients who do not carry the BAFF-var allele, those carrying the allele displayed increased disease activity at the time of study entry and more frequently required intensive treatment with immunosuppressive agents.

Previous studies demonstrated that the frequency of the BAFF-var allele was higher among SLE patients from various European cohorts than among the corresponding control populations, a finding suggesting a relationship between the BAFF-var and SLE [17, 19]. The frequency of BAFF-var allele carriers (9.2%) and the MAF of the BAFF-var allele (4.6%) identified in our SLE study cohort were in line with and support the findings of case-control studies performed by González-Serna et al. [19]. We detected the BAFF-var allele in 7.1% of SLE patients in our German cohort and the corresponding MAF was 3.69%. In comparison, the frequency of the BAFF-var allele (4%) and the MAF (2.02%) among the healthy German population were markedly lower. Similarly, the prevalence of the BAFF-var allele among healthy control subjects from another northern country (the Netherlands) was 3.8%, and the MAF was 1.91%. Among healthy subjects from Spain, the frequency (8.8%) and MAF (4.2%) of the BAFF-var allele were increased; however, among the corresponding Spanish SLE cohort the prevalence and MAF of the BAFF-var allele were even higher than in healthy control subjects with 10.8 and 5.81%, respectively [19]. These variations are mainly due to the previously described north-south gradient underlying the higher minor allele frequency of the BAFF-var allele observed for southern European countries (Spain, 4.2%; Italy, 5.9%) than for the northern European countries (United Kingdom, 1.8%; Netherlands, 1.9%; Germany, 2.02%) [17, 19]. No homozygous carries of the BAFF-var allele were identified in our SLE cohort. The finding is in line with observations of the group of González-Serna et al. who did not detect any homozygotes within the control cohorts from Germany and Netherlands and identified only one homozygous carrier of the BAFF-var allele among 427 German SLE patients [19]. Related to the low allele frequency of BAFF-var in Northern Europe the appearance of carriers with homozygous genotype is very rare. It is noteworthy that our study population consisted predominantly of SLE patients from Switzerland rather than patients representing a central-European population influenced by migration. Hence, a low percentage of these SLE patients were of African or Asian ancestry. These facts should be considered when the results of our study are interpreted, because they may explain why the prevalence of the BAFF-var allele in our cohort is slightly higher compared to the German SLE cohort reported by González-Serna et al. [19]. However, separate analysis of the prevalence of the BAFF-var allele among German SLE patients from the University of Essen and patients from the SSCS found that both subgroups exhibited the same MAF (4%) (data not shown). A limitation of our study is a missing second control population from Switzerland, however we assumed a comparable genetic Caucasian background in both study cohorts.

We focused primarily on analyzing the association of the BAFF-var genotype with several disease manifestations of SLE. Here, our data contradict the observations of Steri et al., who found no association between the BAFF-var allele and clinical manifestations of SLE in 302 patients from the Sardinian SLE population [17]. In contrast, we demonstrated for the first time an association between the BAFF-var allele and SLE disease activity and renal involvement. However, the analysis of overall 362 Italian SLE patients performed by Steri et al. revealed a trend towards increased occurrence of renal disorders among SLE patients with the BAFF-var genotype compared to those without supporting our data [17]. These findings are likely attributable to the fact that the presence of the BAFF-var allele led to an increased production of soluble BAFF, thereby resulting in sustained high levels of BAFF. Consistently, several studies have found that elevated BAFF levels are linked to lupus nephritis and exacerbation of SLE [11–14]. However, serum concentrations of BAFF are modulated by additional regulatory factors (e.g., current B-cell number, type of immunosuppressive treatment etc.) and may vary widely. In contrast, the BAFF-var allele is inherited and its expression does not depend on current therapies or other factors potentially influencing serum levels. Thus, the presence of BAFF-var allele constitutively impairs the downregulation of BAFF expression and results in a predisposition to higher levels of BAFF, which are implicated in the immunopathological pathway of SLE and influence the course of disease among carriers of the BAFF-var allele. A previous study of promoter and exon polymorphisms of the BAFF gene [23] failed to show causality with BAFF synthesis and demonstrated in general only a weak association with susceptibility to SLE. However, this study did not analyze the BAFF-var allele [23].

Interestingly, the effect of the BAFF-var allele was restricted to the lupus nephritis without heightening the frequency of other organ involvements. We hypothesize that occurrence of the BAFF-var allele might induce the intrarenal overexpression of BAFF besides increasing the levels of soluble BAFF in peripheral circulation. In respect to latter studies, release of BAFF by renal tubular epithelial cells was described to contribute to the pathogenesis of lupus nephritis in mice and humans [24–26]. Schwarting et al. showed that BAFF derived from renal tubular epithelial cells activates production of colony forming factor 1 via autocrine loop by binding to the BAFF receptors located on tubular epithelial cells resulting in macrophage influx and creating an inflammatory microenvironment in the affected kidneys [24]. Additionally, local constitutive expression of BAFF within the kidney promotes formation of large organized tertiary lymphoid organs in lupus nephritis leading to expansion of activated B cells and plasmablasts reactive to renal antigens [25, 26]. Otherwise, elevated intrarenal BAFF production mediates glomerular damage in case of lupus nephritis by inducing invasion of T cells secreting proinflammatory cytokines as well as attracting Th17 cells [26]. Moreover, local production of BAFF by infiltrating immune cells retains a proliferative effect on glomerular mesangial cells through interaction with its receptor BAFF-R following activation of protein kinase B and consequently stimulating the development of lupus nephritis [27].

Our results imply that it may be reasonable to stratify SLE patients according to their BAFF-var allele status as a genetic risk factor. On the other hand, we are aware of the BAFF-var allele being a rare genetic variant and occurring at a relatively low allele frequency. Moreover, besides the presence of the BAFF-var allele and high serum levels of BAFF, a range of other factors exert an effect on the course of SLE [5, 6]. However, we believe that the BAFF-var allele is of high clinical significance as a promising candidate for a genetic risk marker that can determine which SLE patients may require extended follow-up care and intensive immunosuppressive therapy because of the relationship between this allele and the risk of severe SLE with renal manifestations.

Moreover, with respect to individualized therapeutic approaches for SLE patients, we assume that knowledge of the patient’s BAFF-var status and subsequent discrimination between patients carrying this allele and those not carrying it may be useful for making the decision about whether to use therapeutic strategies based on anti-BAFF. B-cell depletion therapies, such as rituximab, may not be appropriate for carriers of the BAFF-var allelic variation because of the potentially rapid restitution of B-cells as a result of high levels of circulating BAFF [28, 29]. Furthermore, the effects of belimumab on SLE patients carrying the BAFF-var allele are controversial. On the one hand, patients with the BAFF-var allele may benefit from early treatment with belimumab, which targets BAFF overexpression. On the other hand, the presence of the BAFF-var allele may lead to a weaker response or even resistance to belimumab, so that higher dosages would be necessary for overcoming the overproduction of BAFF. Therefore, we assume that the response to belimumab among carriers of the BAFF-var allele and appropriate control subjects, as well as the detection of the BAFF-var allele among patients who do not respond to belimumab, warrants investigation in future studies.

Of importance, there are some limitations to our study. Our cohort was small in comparison to those involved in previously reported studies of the rare BAFF-var allele and SLE. Also, we did not include a control population from Switzerland; however, we anticipated that both study cohorts would exhibit a comparable genetic (white) background. We analyzed the association between BAFF-var allele status and disease activity scores (SLEDAI and PGA) as unstable variables that changed, in particular with regard to dependence on therapy. To avoid bias, we decided to use exclusively the SLEDAI and PGA scores captured at the time of entry into the SSCS or into the SLE cohort from the University Hospital Essen. The time points of diagnosis and study entry were congruent at least in one third of the 195 SLE patients in our cohort. Otherwise, in consistence with data on the association between the BAFF-var allele and disease activity at study entry, patients with the BAFF-var allele received more-intensive treatment for SLE, with a significantly higher frequency of immunosuppressant administration.

## Conclusions

Our study revealed a striking association of BAFF-var allele with the occurrence of lupus nephritis, renal activity markers such as proteinuria and microhematuria and disease activity defined by the SLEDAI and PGA at study entry. Consequently, carriers of the BAFF-var allele required more frequently immunosuppressive treatment than those tested negative for BAFF-var. Therefore, determining the BAFF-var status of SLE patients may improve the risk stratification of patients for whom the development of lupus nephritis is more likely and thus may be helpful in the follow-up care and treatment of SLE patients.

## Data Availability

All data generated or analyzed during this study are included in this published article.

## Abbreviations

ANA: Antinuclear antibodies
BAFF: B-cell activating factor
CI: Confidence interval
CNS: Central nervous system
dsDNA: Double-stranded deoxyribonucleic acid
MAF: Minor allele frequency
mRNA: Micro ribonucleic acid
NF90: Nuclear factor 90
OR: Odds ratio
PCR: Polymerase chain reaction
PGA: Physician’s global assessment
SLE: Systemic lupus erythematosus
SLEDAI: Systemic Lupus Erythematosus Disease Activity Index
SSCS: Swiss SLE Cohort Study
TNFSF13B: Tumor necrosis factor ligand superfamily member 13b

## References

[1] Mirabelli G, Cannarile F, Bruni C, Vagelli R, De Luca R, Carli L (2015). One year in review 2015: systemic lupus erythematosus. Clin Exp Rheumatol.

[2] Korbet SM, Schwartz MM, Evans J, Lewis EJ, Collaborative Study Group (2007). Severe lupus nephritis: racial differences in presentation and outcome. J Am Soc Nephrol.

[3] Sherer Y, Gorstein A, Fritzler MJ, Shoenfeld Y (2004). Autoantibody explosion in systemic lupus erythematosus: more than 100 different antibodies found in SLE patients. Semin Arthritis Rheum.

[4] Rahman A, Isenberg DA (2008). Systemic lupus erythematosus. N Engl J Med.

[5] Gualtierotti R, Biggioggero M, Penatti AE, Meroni PL (2010). Updating on the pathogenesis of systemic lupus erythematosus. Autoimmun Rev.

[6] Pan Q, Chen J, Guo L, Lu X, Liao S, Zhao C (2019). Mechanistic insights into environmental and genetic risk factors for systemic lupus erythematosus. Am J Transl Res.

[7] Kandala NB, Connock M, Grove A, Sutcliffe P, Mohiuddin S, Hartley L, et al. Belimumab: a technological advance for systemic lupus erythematosus patients? Report of a systematic review and meta-analysis. BMJ Open. 2013;3. 10.1136/bmjopen-2013-002852. 10.1136/bmjopen-2013-002852 PMC3717447 23872289

[8] Bangert E, Wakani L, Merchant M, Strand V, Touma Z (2019). Impact of belimumab on patient-reported outcomes in systemic lupus erythematosus: review of clinical studies. Patient Relat Outcome Meas.

[9] Vincent FB, Morand EF, Schneider P, Mackay F (2014). The BAFF/APRIL system in SLE pathogenesis. Nat Rev Rheumatol.

[10] Sanchez-Niño MD, Ortiz A (2015). That obscure object of Desire': in systemic lupus erythematosus B-cell activating factor/B-lymphocyte stimulator is targeted both by the immune system and by physicians. Nephrol Dial Transplant.

[11] Friebus-Kardash J, Branco L, Ribi C, Chizzolini C, Huynh-Do U, Dubler D, Roux-Lombard P, Dolff S, Kribben A, Eisenberger U, Trendelenburg M (2018). Immune complexes containing serum B-cell activating factor and immunoglobulin G correlate with disease activity in systemic lupus erythematosus. Nephrol Dial Transplant.

[12] Vincent FB, Northcott M, Hoi A, Mackay F, Morand EF (2013). Association of serum B cell activating factor from the tumour necrosis factor family (BAFF) and a proliferation-inducing ligand (APRIL) with central nervous system and renal disease in systemic lupus erythematosus. Lupus.

[13] Petri M, Stohl W, Chatham W, McCune WJ, Chevrier M, Ryel J (2008). Association of plasma B lymphocyte stimulator levels and disease activity in systemic lupus erythematosus. Arthritis Rheum.

[14] Elbirt D, Asher I, Mahlab-Guri K, Bezalel-Rosenberg S, Edelstein V, Sthoeger Z (2014). BLyS levels in sera of patients with systemic lupus erythematosus: clinical and serological correlation. Isr Med Assoc J.

[15] Blair HA, Duggan ST (2018). Belimumab: a review in systemic lupus erythematosus. Drugs.

[16] Teng YKO, Bruce IN, Diamond B, Furie RA, van Vollenhoven RF, Gordon D (2019). Phase III, multicentre, randomised, double-blind, placebo-controlled, 104-week study of subcutaneous belimumab administered in combination with rituximab in adults with systemic lupus erythematosus (SLE): BLISS-BELIEVE study protocol. BMJ Open.

[17] Steri M, Orrù V, Idda ML (2017). Overexpression of the cytokine BAFF and autoimmunity risk. N Engl J Med.

[18] Idda ML, Lodde V, McClusky WG (2018). Cooperative translational control of polymorphic BAFF by NF90 and miR-15a. Nucleic Acids Res.

[19] González-Serna D, Ortiz-Fernández L, Vargas S (2018). Association of a rare variant of the TNFSF13B gene with susceptibility to rheumatoid arthritis and systemic lupus erythematosus. Sci Rep.

[20] Castrejón I, Tani C, Jolly M, Huang A, Mosca M (2014). Indices to assess patients with systemic lupus erythematosus in clinical trials, long-term observational studies, and clinical care. Clin Exp Rheumatol.

[21] Ceccarelli F, Perricone C, Massaro L, Cipriano E, Alessandri C, Spinelli FR (2015). Assessment of disease activity in systemic lupus erythematosus: lights and shadows. Autoimmun Rev.

[22] Miller SA, Dykes DD, Polesky HF (1988). A simple salting out procedure for extracting DNA from human nucleated cells. Nucleic Acids Res.

[23] Kawasaki A, Tsuchiya N, Fukazawa T, Hashimoto H, Tokunaga K (2002). Analysis on the association of human BLYS (BAFF, TNFSF13B) polymorphisms with systemic lupus erythematosus and rheumatoid arthritis. Genes Immun.

[24] Schwarting A, Relle M, Meineck M, Föhr B, Triantafyllias K, Weinmann A (2018). Renal tubular epithelial cell-derived BAFF expression mediates kidney damage and correlates with activity of proliferative lupus nephritis in mouse and men. Lupus.

[25] Sun CY, Shen Y, Chen XW, Yan YC, Wu FX, Dai M (2013). The characteristics and significance of locally infiltrating B cells in lupus nephritis and their association with local BAFF expression. Int J Rheumatol.

[26] Kang S, Fedoriw Y, Brenneman EK, Truong YK, Kikly K, Vilen BJ (2017). BAFF induces tertiary lymphoid structures and positions T cells within the glomeruli during lupus nephritis. J Immunol.

[27] Zheng N, Wang D, Ming H, Zhang H, Yu X (2015). BAFF promotes proliferation of human mesangial cells through interaction with BAFF-R. BMC Nephrol.

[28] Dass S, Rawstron AC, Vital EM, Henshaw K, McGonagle D, Emery P (2008). Highly sensitive B cell analysis predicts response to rituximab therapy in rheumatoid arthritis. Arthritis Rheum.

[29] Juge PA, Gazal S, Constantin A, Mariette X, Combe B, Tebib J (2017). Variants of genes implicated in type 1 interferon pathway and B-cell activation modulate the EULAR response to rituximab at 24 weeks in rheumatoid arthritis. RMD Open.

